# A Case of Ichthyosis Vulgaris and the Use of 70% Glycolic Acid Chemical Peels for Management

**DOI:** 10.7759/cureus.29334

**Published:** 2022-09-19

**Authors:** Victoria Palmer, Patricia Dunwell

**Affiliations:** 1 Dermatology, Musgrave Medical Centre, Kingston, JAM

**Keywords:** salicylic acid, beta hydroxy acid, alpha hydroxy acid, cosmetic dermatology, general dermatology, glycolic acid, chemical peels, common ichthyosis, atopic dermatitis, ichthyosis vulgaris

## Abstract

Ichthyosis vulgaris is the most common of the inherited ichthyoses, with a semi-autosomal dominance. Approximately 37-50% of people affected have associated atopic eczema and a similar proportion have atopic relatives. In this case report, we present a 15-year-old female with a history of atopic eczema who clinically presented with asymptomatic, brown, scaly patches on the extensor surfaces of the lower limbs and flexural surfaces of the upper limbs, sparing the flexural creases on all limbs. She also had neck, abdominal, and mild truncal involvement. A clinical diagnosis of ichthyosis vulgaris was made and the differential diagnoses of X-linked recessive ichthyosis, pityriasis rubra pilaris, and xerotic dermatitis were entertained. While waiting for the histological report, she was maintained on 5% glycerine in aqueous cream for daily moisturizing with alternating nightly applications of 10% glycolic acid lotion and a 5% salicylic acid mixture. The diagnosis of ichthyosis vulgaris was confirmed by histopathological findings of hyperkeratosis and an absent granular layer. She then received a 70% glycolic acid in-office chemical peel on the abdomen to test for the cosmetic outcome. The glycolic acid peel was approximately 90% efficacious in reducing the hyperkeratinization and is recommended as an adjunctive biannual maintenance regime in combination with other topical therapies such as daily emollients, topical alpha/beta hydroxy, and/or urea compounds.

## Introduction

Ichthyosis vulgaris (IV) is prevalent in 1 in 80 to 1 in 250 individuals, more common in temperate climates, and has an equal sex distribution [1-3]. Approximately 37-50% of people affected have associated atopic eczema and a similar proportion have atopic relatives [1]. In the literature, it is commonly treated with humectants, low-grade keratinolytic therapy, and, occasionally, systemic retinoids. The aim of this report is to demonstrate the benefit of glycolic acid chemical peels for the management of ichthyosis vulgaris, especially given the context of a background of atopic dermatitis and the lack of available information in the literature.

## Case presentation

**Figure 1:**
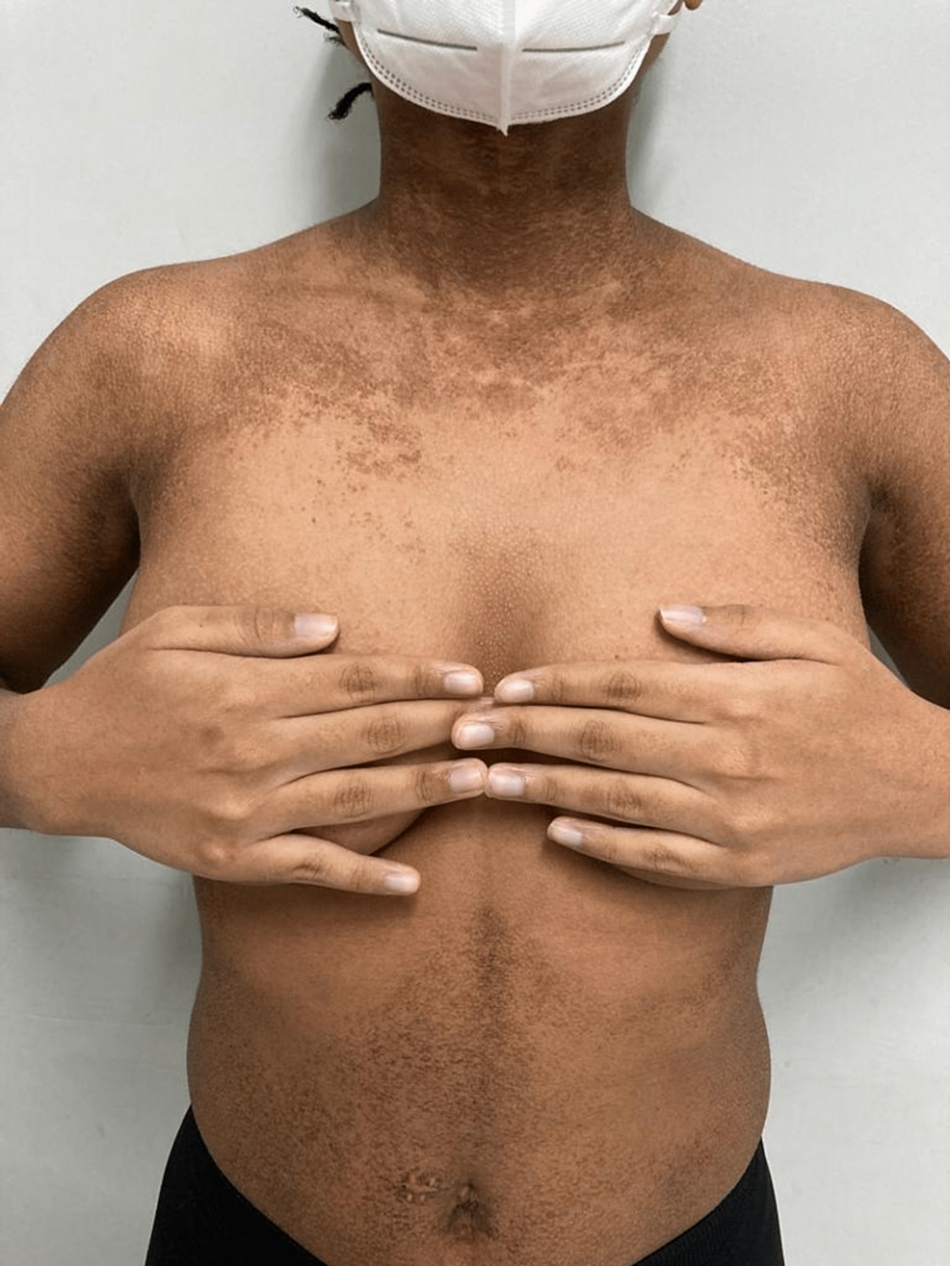
Hyperpigmented patches and scaling on the neck, upper trunk, and abdomen

**Figure 2:**
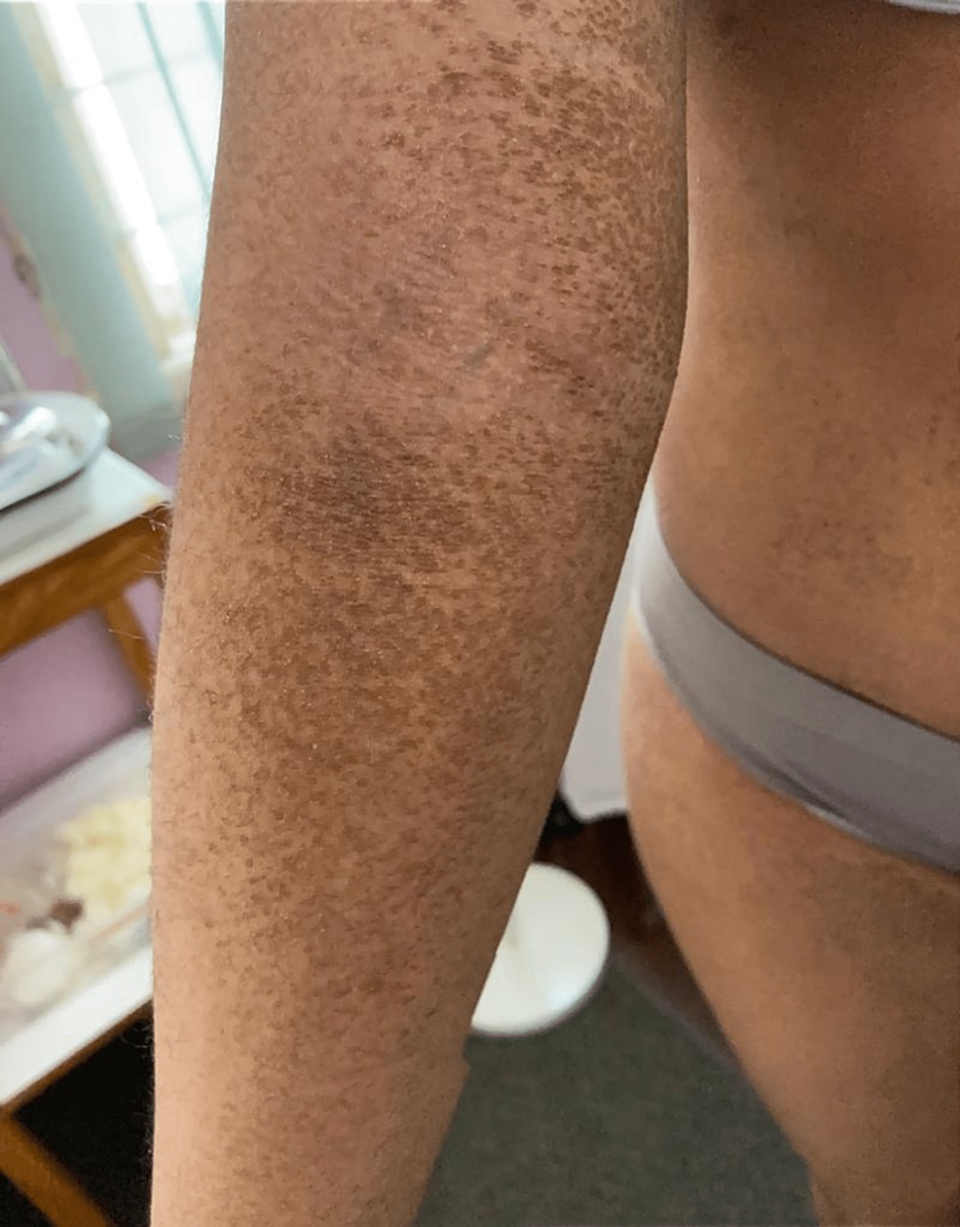
Hyperpigmentation and scaling of the right upper limb with sparing of the flexural creases

We present a 15-year-old, female student with a history of atopic eczema of the flexures and dyshidrotic eczema of the right middle and index fingers, who presented with persistent asymptomatic discoloration of the neck and body for 10 months. On examination, the skin was xerotic with gross hyperpigmentation and scaling noted to the neck, upper trunk, limbs, and abdomen. On the limbs, predominantly, the ventral surface of the upper limb and the dorsal surface of the lower limb were involved, with sparing of the back and flexural creases (Figures 1-2).

Additionally, follicular accentuation was noted in the lateral thighs. A biopsy of the abdominal skin was performed, and the patient was managed with 5% glycerine in aqueous cream PRN and 5% salicylic acid (SA) cream alt 10% glycolic acid (GA) nocte, pending review of the biopsy results. The biopsy report subsequently described hyperkeratosis, papillomatosis, an absent granular layer, a sparse superficial perivascular lymphocytic infiltrate, and melanophages, with periodic-acid-Schiff (PAS) stain negative for fungal elements. These findings were consistent with IV, with differential diagnoses, including other causes such as pityriasis rotunda, X-linked recessive ichthyosis, pityriasis rubra pilaris, and xerotic dermatitis.

**Figure 3:**
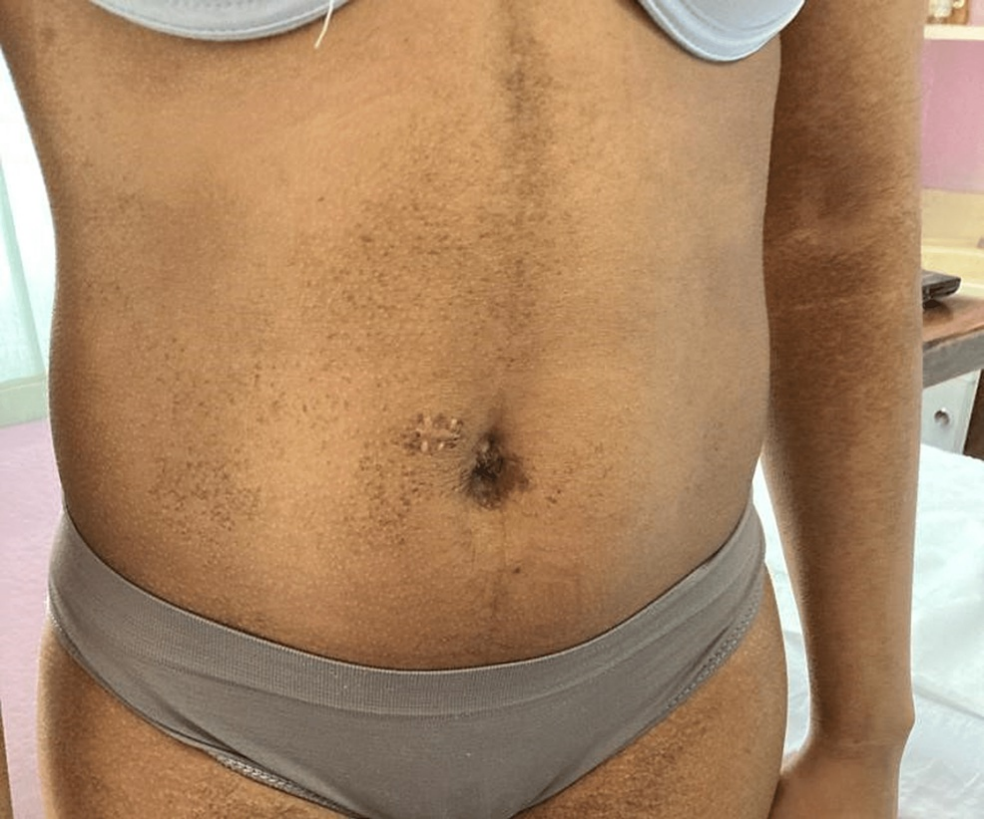
Abdomen post 70% glycolic acid chemical peel

At the one-month review, the patient acknowledged improvement in the form of moisture retention and a slight decrease in pigmentation in the affected areas. At this time, a GA 70% peel was done to the abdomen, after which she was instructed to apply 1% hydrocortisone cream and 5% glycerine in aqueous cream for 2/52 before transitioning to alternate 10% GA/5% SA mixtures nocte. In the one-week review post-procedure, the abdomen was noted to be significantly (90%) improved (Figure 3).

A chemical peel was then performed on other affected areas after one month and a biannual maintenance regime of 70% GA peels was agreed upon.

## Discussion

The currently recommended first, second, and third-line treatments for IV are summarized in Table 1 [1].

**Table 1:** Treatment options in the literature for IV Beta-hydroxy acids (BHAs) can include 60% propylene glycol, water, and alcohol gel containing 6% SA. AHAs include options such as 5-10% lactate, glycolic, mandelic, and malic acids. *Systemic and topical retinoids not advocated: These may exacerbate ichthyosis so are advised more for other disorders of cornification such as lamellar ichthyosis, congenital ichthyosiform erythroderma, and palmoplantar keratoderma.

First Line	Advise of chronicity, ensure hydration (humidification, bathing) and lubrication (lotions, creams, ointments)
Second Line	Keratinolytics (AHAs> BHAs/ 5-10% urea/12% ammonium lactate), topical retinoids (tretinoin, tazarotene), topical vitamin D (calcipotriol)
Third Line	Systemic retinoid therapy* (isotretinoin/acitretin)

The diagnosis of IV is reasonably deduced from history and physical examination, as seen in Table 2, but a biopsy (to reveal orthohyperkeratosis, absent/reduced granular layer) and genetic testing (FLG mutation) are useful diagnostic adjuncts as well.

**Table 2:** Outline of diagnostic tools for IV * IV is characteristically more pronounced on the extensor surfaces of arms and legs and spares the flexural creases, and the trunk is mildly involved with the sparing of the diaper area.

Physical Examination	History
Fine white/grayscale with central attachment, typical distribution*	Onset is usually in early childhood
Palmer hyper linearity	Improvement with warm weather and emollients
Keratosis pilaris	Familial occurrence
	Atopic diathesis
	Possible improvement with age

The etiology of IV is multifactorial, with primary associations made among the absence of profilaggrin (leading to a loss of function of FLG), decreased expression of filaggrin messenger RNA, and possibly increased adhesiveness of the stratum corneum cells or failure of normal separation, resulting in changes in cell adhesion at desmosomes [1-2,4].

GA is the smallest and most aggressive alpha hydroxy acid (AHA). This characteristic is necessary for the lysis and subsequent shedding of more superficial, less hydrated corneocytes, which seem to be less sensitive to the action of AHAs than the corneocytes at the base of the stratum corneum [4]. It, therefore, produces a more compact stratum corneum, which helps with the retention of moisture in the dermal layer. Additionally, it increases glycosaminoglycan deposition and builds collagen and elastin over a period of time [5]. These properties, therefore, make GA ideal for hyperkeratinization disorders, even in patients with atopic diatheses.

Beta-hydroxy acids (BHAs), such as SA, are lipophilic and have keratolytic and comedolytic properties, which allows for greater penetration of comedones. They may, therefore, be more useful for comedonal acne [5-6]. However, future comparative studies would be helpful in contrasting the different results for this condition.

One concern regarding the use of chemical peels on patients prone to atopic eczema is that it may aggravate the latter. One rule of thumb would be not to exfoliate the skin if there are any signs of irritated skin or active dermatitis. The use of emollients is also emphasized. Another concern may be the short-lasting nature of the results. However, these authors have found chemical peels to be useful as a biannual maintenance regime. They may act as an adjunct to urea or low-percentage AHAs and/or BHAs, the latter of which have decreased efficacy with time [5].

Further research involving a larger sample size is needed to reinforce the outcomes of this study, and the authors intend to publish a case series for this purpose.

Keyword search terms “ichthyosis vulgaris”, “dry skin”, “common ichthyosis”, “scaly skin”, “xerosis”, “glycolic acid”, “salicylic acid”, and “chemical peel*” were reviewed on OVID Medline, PubMed, COCHRANE Database of Systematic Reviews, Latin America and Caribbean Health Sciences Literature (LILACS), and Web of Science (WOS), with no restrictions applied to year or publication status. Of the 1295 articles reviewed after duplicates were removed, zero studies were included in the meta-analysis.

## Conclusions

The literature lacks information regarding the use of chemical peels in IV. This report demonstrates that the use of 70% glycolic acid chemical peels is, however, an effective tool for the removal of an excessively keratinized epidermis. Additionally, it may serve as a biannual maintenance regime for people with ichthyosis vulgaris. Other components necessary for the maintenance regime include the daily use of humectants and chemical exfoliants, such as urea and low-percentage BHA and AHs, respectively. This report also highlights that high-percentage chemical peels can be performed safely in people with concomitant atopic dermatitis, under the close supervision of a physician, as the mechanism of action promotes a compact stratum corneum and helps with moisture retention in the dermis.
